# Stable myoelectric control of a hand prosthesis using non-linear incremental learning

**DOI:** 10.3389/fnbot.2014.00008

**Published:** 2014-02-25

**Authors:** Arjan Gijsberts, Rashida Bohra, David Sierra González, Alexander Werner, Markus Nowak, Barbara Caputo, Maximo A. Roa, Claudio Castellini

**Affiliations:** ^1^IDIAP Research InstituteMartigny, Switzerland; ^2^Robotics and Mechatronics Center, German Aerospace Center (DLR)Weßling, Germany

**Keywords:** surface electromyography, machine learning, incremental learning, human–machine interfaces, rehabilitation robotics, force control

## Abstract

Stable myoelectric control of hand prostheses remains an open problem. The only successful human–machine interface is surface electromyography, typically allowing control of a few degrees of freedom. Machine learning techniques may have the potential to remove these limitations, but their performance is thus far inadequate: myoelectric signals change over time under the influence of various factors, deteriorating control performance. It is therefore necessary, in the standard approach, to regularly retrain a new model from scratch. We hereby propose a non-linear incremental learning method in which occasional updates with a modest amount of novel training data allow continual adaptation to the changes in the signals. In particular, Incremental Ridge Regression and an approximation of the Gaussian Kernel known as Random Fourier Features are combined to predict finger forces from myoelectric signals, both finger-by-finger and grouped in grasping patterns. We show that the approach is effective and practically applicable to this problem by first analyzing its performance while predicting single-finger forces. Surface electromyography and finger forces were collected from 10 intact subjects during four sessions spread over two different days; the results of the analysis show that small incremental updates are indeed effective to maintain a stable level of performance. Subsequently, we employed the same method on-line to teleoperate a humanoid robotic arm equipped with a state-of-the-art commercial prosthetic hand. The subject could reliably grasp, carry and release everyday-life objects, enforcing stable grasping irrespective of the signal changes, hand/arm movements and wrist pronation and supination.

## 1. Introduction

Surface electromyography (sEMG from now on) was introduced in the 1950's and 1960's as a coarse threshold-based control signal to open and close a single-degree-of-freedom (DOF) gripper-like hand prosthesis. This control scheme was designed to restore at least the most basic functionality of the lost limb (Battye et al., 1955; Bottomley, 1965) and its application has proved to be safe. In short, the activity of (remnant) muscles is measured using sEMG electrodes and the magnitude of these signals is used to determine how fast the gripper should open or close. No more than two electrodes are used, one for the opening movement and one for the closing movement. This simple threshold-based schema is still in use in the majority of commercially available prostheses. Back in 1967, however, Finley and Wirta (1967) used a linear pattern-matching algorithm to better understand the intent of the amputee. This inspired a new strand of research aimed at enriching the possibilities offered to the patient. The idea was that myoelectric signals could be used to detect whether the subject wanted to do a pinch grip, a power grasp, and so on. With the recent advent of multi-fingered prostheses (e.g., Otto Bock's *Michelangelo* and Touch Bionics's *i-LIMB*), the need for more dexterous control has become even stronger. As is evident from, for instance, the review by Peerdeman et al. (2011), a vast variety of machine learning methods have been proposed toward this goal, often to an excellent degree of precision in controlled laboratory conditions.

Despite these advancements, the clinical and practical application of these techniques is still lacking; this is at least partially due to the limited flexibility of the employed pattern recognition systems to deal with the inherent non-stationarity of sEMG. Myoelectric signals are known to change over time under the influence of muscle fatigue, changing conductivity (e.g., perspiration, humidity, or temperature), electrode displacement, or even differences in the patterns produced by the user. Moreover, changes in the position and velocity of the arm influence the signal (Fougner et al., 2011). With batch learning methods, used in the majority of studies, this signal shift and unanticipated variability inevitably leads to performance degradation and the need to retrain a new model from scratch in many possible different conditions.

Among the first to address the problem of performance degradation were Nishikawa et al. (2000), who implemented a supervised adaptation technique that allowed subjects to manually correct unsatisfactory predictions. This is in contrast to the unsupervised techniques by Kato et al. (2006) or Chen et al. (2013), in which the learning method provides itself feedback by treating selected samples and its corresponding predictions as additional training data. A compelling advantage of unsupervised adaptation is that it obviates the need for manual intervention by the user; however, self-training strategies are susceptible to reinforcing their own classification mistakes, particularly in case of abrupt distribution shifts. A comparison by Sensinger et al. (2009) indeed demonstrates that unsupervised adaptation methods are less effective at avoiding performance degradation than supervised alternatives.

All these studies considered a *sequential movement classification* setting, where a classifier is trained to predict from a discrete and predefined set of hand postures. However, *proportional* control of multiple (DOFs) has recently gained momentum (see Fougner et al., 2012 and references therein) as it allows much more fine-grained control of the prosthesis. To the best of our knowledge, only two studies have discussed performance degradation in the proportional control setting. Artemiadis and Kyriakopoulos (2011) propose a switching-regime approach to control the Cartesian position of a robotic arm using sEMG. A shortcoming with this approach is that changes to the myoelectric signals need to be present during the initial training of the regimes. This requirement increases initial training time considerably and it seems doubtful whether all sources of variation can be sufficiently anticipated (cf. perspiration). In other recent work, Pilarski et al. (2011) learned a control policy for a two-DOFs robot arm using Actor-Critic Reinforcement Learning. Their experiments show that control policies can be learned successfully even when the human provides sparse positive and negative rewards. This human-driven feedback mechanism also allows users to encourage the system to adapt its policy to (slight) distribution changes. However, the approach requires that reward or feedback is applied consistently. Failing to provide positive reward when the system is performing satisfactory, for instance, may slowly degrade a previously stable policy.

In this work, we instead propose to use a supervised incremental learning method to predict finger forces and graded-force grasping patterns from sEMG. The idea is that a user could perform a quick update when performance degrades to let the model adapt to distribution changes, without the need for costly retraining from scratch. The algorithm we used was an incremental variant of Ridge Regression (RR; Hoerl and Kennard, 1970), supporting computationally efficient updates of the regression model when new training samples arrive. This algorithm has recently been used to predict finger forces using ultrasound (Sierra González and Castellini, 2013); however, in contrast to their high-dimensional image features (Castellini et al., 2012), linear models are typically not sufficient to model the relationship between sEMG and finger forces (Gijsberts et al., 2014), especially when using a reduced, low-resolution set of commercially available sEMG electrodes. We therefore adopted the approach by Gijsberts and Metta (2011), who combined incremental RR with Random Fourier Features (RFFs) (Rahimi and Recht, 2008b) to allow use on non-linear problems. The resulting algorithm, subsequently referred to as incremental Ridge Regression with Random Fourier Features (iRFFRR), has been shown to attain excellent generalization performance in the robotics application domain even with a relatively modest number of training samples (Gijsberts and Metta, 2011; Droniou et al., 2012). Furthermore, predictions as well as model updates require a constant amount of computational resources regardless of the number of updates, thus allowing use in a hard real-time setting (e.g., on embedded hardware). A final advantage is that multiple output dimensions can be learned simultaneously at negligible additional cost. This approach is therefore particularly well suited for the *simultaneous and proportional* control setting (as opposed to discrete and sequential) advocated by Farina and others (Jiang et al., 2012).

The effectiveness of iRFFRR has been assessed in two experiments. The first experiment, which we will call *Algorithm evaluation*, dealt with the effectiveness in countering the medium-term changes in the sEMG signal: the method was used to predict single-finger forces recorded with an accurate force sensor, using sEMG gathered across a relatively long time (2 days, with electrode removal and re-positioning in-between days). We also tested the method's performance when trained on visual stimulus values rather than sensor values, and when trained on minimal and maximal forces only. This represents a realistic setting, since amputees cannot operate any force sensor whatsoever and can hardly perform any graded-force task to calibrate the system, as they lack proprioception and sometimes suffer from an inconsistent phantom-limb feeling.

The second experiment, which we will call *Demonstration*, was an on-line teleoperation task in which iRFFRR enabled an intact human subject to grasp, carry and release everyday-life objects. A standard magnetic tracker placed on the subject's wrist was used to control the position of the robotic end effector; as end-effector, we used a commercially available *i-LIMB Ultra* prosthetic hand by Touch Bionics. On the other hand, each finger motor was controlled using iRFFRR applied to sEMG. The subject repeatedly performed the required tasks with a stable grasp, notwithstanding the inevitable motion of arm/hand and the pronation/supination of the wrist.

## 2. Materials and methods

### 2.1. Experimental setup

The experimental setup consisted of four main elements. For the first experiment, we used an array of 10 sEMG electrodes to gather the sEMG signal from the subjects' forearms and a force sensor to record fingertip forces. For the second experiment, the same array of sEMG electrodes was used, plus we employed a magnetic position tracker in order to track the position and orientation of the subject's wrist, to be used to control the position of the end-effector of the slave platform during the teleoperation. The slave setup was the TORO humanoid platform developed at the DLR.

#### 2.1.1. Surface electromyography

**Figure 1:**
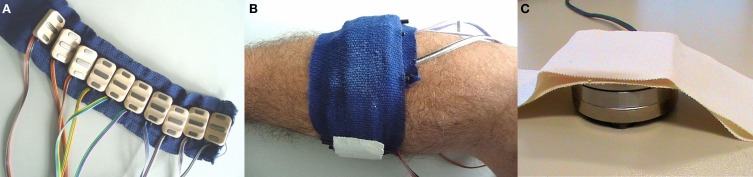
**The setup of the first experiment. (A)** Ten OttoBock MyoBock 13E200 sEMG electrodes, uniformly arranged **(B)** on the subject's forearm using a band of bio-compatible adhesive tape. **(C)** An ATI Mini45 force sensor, kept in place using double-sided tape.

Ten OttoBock *MyoBock 13E200* sEMG electrodes (www.ottobock.com) were used to capture muscle activations from the forearm. These electrodes provide an amplified, bandpass filtered, rectified sEMG signal, eliminating the need of further external signal conditioning; they are the standard, off-the-shelf sEMG device used in clinical prosthetic sockets, and are commercially available. The electrodes were arranged on a uniformly-spaced band of bio-compatible reusable adhesive tape. This uniform electrode positioning has already been demonstrated to be effective in combination with machine learning methods, even on amputees (Castellini et al., 2009). Figure 1 shows (A) the electrodes arranged on the band and (B) the placement of the band on a subject's forearm.

The band was fixed to the subject's forearm approximately 7cm below the elbow. The position of the electrodes was always maintained fixed on the elastic band. A pencil mark indicating the position of one of the electrodes was drawn on the subject's forearm to be able to reposition the band in the same location in subsequent sessions, whenever required.

The sensor values were recorded at 100 Hz using a DAQ card. This sampling rate does not conflict with the spectrum of myoelectric signals (i.e., roughly 5–500 Hz), since bandpass filtering and rectification is already done on-board the electrodes prior to acquisition by the DAQ card. The effective spectrum of this rectified signal is well within the 50 Hz frequency limit supported by our acquisition setting.

#### 2.1.2. Fingertip forces

A single ATI Mini45 SI-290-10 force sensor (www.ati-ia.com) was employed to capture the force exerted by each finger in turn. This sensor guarantees a linear output and has a resolution of 0.125N. The sensor was taped onto the setup table at a convenient distance from the subject's hand, so that minimal movement was required to press the sensor with any finger. The sensor was connected to a DAQ card on a dedicated computer and its measurements were broadcast over a local network using a UDP stream. Figure 1C shows the sensor, taped to the setup table.

#### 2.1.3. Position tracking

A Polhemus FASTRAK magnetic tracker (www.polhemus.com) was employed to track the subject's wrist position and orientation. Using an oscillating magnetic field, this device returns, at a rate of 130 Hz, the position and orientation of a small sensor which can be conveniently placed on the human body by means of bio-compatible tape or velcro. The static tracking accuracy of this device is 0.0762cm (position) and 0.15° (orientation). The range of motion is 4.6m. The device was initially tested to check that these values would hold in practice, and would be suited to carry the experiment on.

#### 2.1.4. Teleoperation setup

The teleoperation slave setup was the TORO (TOrque controlled RObot), a full-body humanoid with 25 DOFs, excluding the hands (Ott et al., 2012). Each leg and arm of this setup has six rotational joints, all equipped with link-side torque sensors, controllable both in torque- and position-control modes. For the present experiment, only the right arm from the shoulder down was used. An off-the-shelf *i-LIMB Ultra* prosthetic hand by Touch Bionics (www.touchbionics.com) was mounted as the right-hand end-effector of the robot. The i-LIMB Ultra is a state-of-the-art poly-articulated hand prosthesis with five active, independently drivable fingers and a manually opposable thumb. The hand was especially fitted with development firmware, allowing direct access to the power electronics driving the five DC motors. Due to the lack of sensors, this hand provides no feedback. The robot was controlled in a hybrid position and force teleoperated fashion, visual feedback being the only cue for the operator.

The finger forces estimated by the method proposed in this paper were scaled and used to directly drive the finger motors, while the robot arm was controlled using Cartesian impedance control to ensure a compliant behavior (Ott et al., 2008). To provide protection against faulty sensor readings of the tracking system, a velocity filter was designed, that sets an upper bound for the translational and rotational velocities. This filtered pose signal is fed to the impedance control, which guarantees that the robot's end-effector follows the trajectory like a spring-damper system. For control of the hand, the predicted finger forces were scaled to the range of ± 7 arbitrary units (a.u.) expected by the prosthesis control system. These set-points were proportionally converted to motor voltages, which in absence of resistance translates directly to rotational velocity of a finger. As soon as a finger comes in contacts with the environment, the applied input voltage is instead directly proportional to the force applied by the finger. The magnitude of these forces are, however, not comparable to human finger forces, as the modular design of the hand (with integrated motors and gears) allows only relatively small forces. These are, however, still sufficient for fine manipulation and power grasping of objects with the help of the friction generated by the rubber surface of the hand.

**Figure 2:**
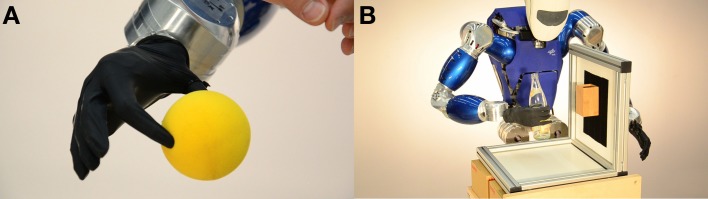
**(A)** The i-LIMB Ultra pinch-gripping a soft foam ball. **(B)** The TORO humanoid robot, grasping a glass bottle.

As a workbench for the teleoperation, a 50 cm wide metal frame with one lateral wall was placed in front of the robot. Velcro straps were attached to the wall to support a wooden container, in which a soft ball could be placed, and a piece of gray foam with a slit to simulate a credit card reader. Additional items that were part of this setup were a glass bottle and a small metal circular support to prevent the soft ball from rolling when placed on the box. Figure 2 shows (A) the prosthetic hand in the act of pinch-gripping a soft foam ball and (B) a typical configuration of the TORO robotic platform, power-grasping and carrying a glass bottle. Additionally, the movie provided in the Supplemental Material shows the teleoperated setup in action.

### 2.2. Non-linear incremental learning

#### 2.2.1. Incremental ridge regression

We start off from an incremental variant of RR, a regularized variant of least-squares regression. RR builds a linear model of the input space data *x* of the form *f*(***x***) = ***w***^T^***x*** (Hoerl and Kennard, 1970). Given a training set of *m* real-valued input–output pairs ***x**_i_* ∈ ℝ^*n*^ and *y*_*i*_ ∈ ℝ, the optimal weight vector ***ŵ*** is defined as

(1) $$\arg\underset{{}_{w}}{\min}\;\frac{\lambda }{2}{\left\|w\right\|}^{2}+\frac{1}{2}\sum_{i=1}^{m}{\left(y_{i}-f\left(x_{i}\right)\right)}^{2},$$

where λ is a hyper-parameter that balances the tradeoff between minimizing the errors and regularizing the solution. This optimization problem reduces to solving a system of *n* linear equations, namely

(2) $$\hat{w}={\left(\lambda I+X^{\text{T}}X\right)}^{-1}X^{\text{T}}y=A^{-1}\beta ,$$

where ***X*** = [***x***_1_, …, ***x**_m_*]^T^ and ***y*** = [*y*_1_, …, *y_m_*]^T^ denote the training inputs and outputs in vector notation.

One of the motivations for using RR in the present work was that it allows incremental updates of the model without the need to store any training samples. Notice, in fact, that the solution in Equation (2) can be decomposed as the product between an inverse covariance matrix ***A***^−1^ and a vector **β**. Since adding a new training sample (***x***_*m* + 1_, *y*_*m* + 1_) corresponds to appending an extra row to both ***X*** and ***y***, it is trivial to update **β** from its previous solution by adding the vector ***x***_*m* + 1_ *y*_*m* + 1_. The inverse covariance matrix ***A***^−1^ can instead be updated by applying the Sherman–Morrison formula, which computes the rank-1 update of the inverse of ***A*** when adding the outer product ***x***_*m* + 1_***x***^T^_*m* + 1_.

Another desirable property of RR is that models for multiple outputs can be learned at negligible additional cost. The primary computational burden in Equation 2 is the inversion of the covariance matrix (or the update thereof), which is not dependent on the output vector ***y***. The same inverse covariance matrix can therefore be used to efficiently solve for multiple output vectors [***y***_1_, …, ***y***_*p*_], assuming that all models share the same input samples ***X***.

#### 2.2.2. Random fourier features

The practical use of RR is limited due to its linearity. In Kernel Ridge Regression (KRR), this limitation is circumvented using the so-called kernel trick (Saunders et al., 1998; Rifkin et al., 2003), which allows the algorithm to be performed implicitly in a potentially infinite dimensional feature space. A consequence of the kernel trick is that the model takes the form of a weighted summation of kernel evaluations with the training samples, that is $f(x)=\sum_{i=1}^{m}c_{i}k(x,x_{i}\text{)}$. Perhaps the most popular kernel function is the well-known Radial Basis Function (RBF) kernel,

(3) $$k\left(x,y\right)=e^{-\gamma {\left\|x-y\right\|}^{2}}\text{ for }\gamma >0,$$

where γ determines the bandwidth of the Gaussian. Though the use of kernels drastically increases the capacity of RR, an adverse effect is that the computational requirements for predictions and incremental updates become dependent on the number of training samples. In other words, the time and memory consumption increase progressively with each incremental update, making kernel RR unsuited for real-time operation. This dependence can be avoided by approximating the kernel function with a finite dimensional feature mapping. Rahimi and Recht (2008a) adopted this strategy and proposed to take a finite number of random samples in the Fourier domain of shift invariant kernel functions (e.g., the RBF kernel). They show that the Random Fourier Feature

(4) $$z_{\omega }\left(x\right)=\sqrt{2}\cos\left(\omega^{\text{T}}x+b\right)$$

produces an unbiased estimate of a kernel if **ω** is drawn from an appropriate distribution μ and if *b* is drawn from a uniform distribution from 0 to 2π. For the RBF kernel with bandwidth γ, the corresponding μ is a normal distribution with covariance 2γ***I***. We will focus exclusively on the RBF kernel in the remainder of this text. The kernel approximation can be made more precise by averaging multiple RFFs *z*_ω_, so that for *D* features we have

(5) $$z\left(x\right)=\frac{1}{\sqrt{D}}{\left[z_{1}\left(x\right),\ldots ,z_{D}\left(x\right)\right]}^{\text{T}},$$

where each feature *z*_*i*_ independently draws an individual **ω**_*i*_ and *b_i_*. To make RR non-linear, it thus suffices to replace each input vector ***x***_*i*_ with its random projection *z*(***x**_i_*). Due to the definition of RFFs, the resulting algorithm will approximate KRR with increasing accuracy as the number of RFFs *D* increases. However, choosing larger *D* comes at the cost of higher computational requirements. Annotated pseudo-code for the complete iRFFRR algorithm is given in Algorithm 1, while the interested reader is referred to the work by Gijsberts and Metta (2011) for a more detailed treatment of the method.

**Algorithm 1:** **Incremental Ridge Regression with RBF Random Fourier Features**.

**Require:** λ > 0, γ > 0, *n* > 0, *D* > 0	
1: *A*^−1^ ← λ^−1^ ***I***_*D* × *D*_	
2: ***w*** ← **0**_*D* × 1_	
3: **β** ← **0**_*D* × 1_	
4: **Ω** ~ 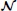 (0, 2 γ)_*D* × *n*_	
5: ***b*** ~ 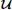 (0, 2π)_*D* × 1_	
6: **for all** (*x, y*) **do**	
7: $z\leftarrow \sqrt{\frac{2}{D}}\cos\text{(}\Omega x+b)$	// *random feature mapping*
8: *ŷ* ← ***w***^T^ ***z***	// *predict label*
9: **β** ← **β** + ***z**y*	
10: $A^{-1}\leftarrow A^{-1}-\frac{A^{-1}zz^{\text{T}}A^{-1}}{1+\;z^{\text{T}}A^{-1}z}$	// *Sherman–Morrison*
11: ***w*** ← ***A***^−1^β	// *update weights*
12: **yield** *ŷ*	
13: **end for**	

### 2.3. Experiment 1: algorithm evaluation

Ten healthy human subjects (age between 23 and 40 years, 8 men and 2 women) were recruited for the algorithm evaluation. Each subject received a thorough description of the experiment, both in oral and written form. Informed written consent was obtained from all participants. Experiments with sEMG and force sensors were approved by the Ethical Committee of the DLR.

#### 2.3.1. Experimental protocol

Each subject sat comfortably on an adjustable office chair, maintaining an upright body posture with both feet on the floor and the elbow bent at approximately 90°; the electrode band was then applied. Prior to the start of the experiment, the maximal voluntary contraction (MVC) was determined for each finger by asking the subject to press the sensor with the largest possible force without feeling discomfort or pain. During the actual experiment, a computer screen in front of the subject showed an animated bar (visual stimulus) for each finger, indicating the required force to be applied on the sensor. More specifically, the participant was required to flex a finger with increasing force until reaching a plateau of 80% maximal voluntary contraction, then continue flexing with a constant force for a small period of time, and finally gradually releasing the force again until reaching the rest level. Notice that the actually produced force values, which were recorded by the force sensor, were *not* displayed in real-time to the subjects. This was done so that the exercise would reflect at least partially the situation of an amputee, who cannot produce any reliable ground truth in principle.

The complete experiment consisted of four *sessions* spread over 2 days, where two sessions were performed per day separated by approximately 5 min of rest. The electrodes were removed after the second session of the first day; on the second day, the pencil mark mentioned in section 2.1.1 was used to place the electrodes roughly in the same position as in the first day. The motivation behind acquiring in multiple sessions and over multiple days was to allow gradual changes within a session or day (e.g., fatigue, temperature adjustment), as well as changes occurring from one day to the other (e.g., electrode repositioning).

Participants were instructed to perform in total 18 *trials* per session, where a trial refers to alternately activating each of the five fingers for a brief duration (starting from the thumb). The stimulus that participants had to match was characterized by a gradual on- and offset that followed a square-sinusoidal pattern, while the plateau in between (i.e., flexing with constant force) had a duration chosen randomly from 1.5, 3, and 4.5 s. After activation of a finger, the participant returned to the rest situation, which lasted for several seconds before activation of the subsequent finger. Since the fingers were not activated simultaneously, the single force sensor was sufficient to record forces for all five fingers (or DOFs) by assuming a zero force for all non-activated fingers. Each of the four sessions thus consisted of 18 × 5 = 90 finger activations and took roughly 20 min.

#### 2.3.2. Feature extraction

We employed the scheme proposed by Englehart and Hudgins (2003), which consists of segmenting the signals in windows, then extracting features from the windows, and finally predicting the output values based on the extracted features. Since the scope of this work is not necessarily to obtain optimal performance, but rather to explore the benefit of incremental learning, we used the established Root Mean Square (RMS) representation within a sliding window of 200 ms length (i.e., 20 samples per window and an increment of 1 sample). The window length was selected during preliminary experiments, and according to suggestions found in related work (Smith et al., 2011). Aside from being easily implemented, a typical argument for RMS features is that it has (under ideal conditions) a quasi- or curvilinear relationship with the force exerted by a muscle (Criswell, 2010). Unfortunately, this linear relationship often does not hold in practical recordings, among other reasons due to muscle cross-talk.

#### 2.3.3. Evaluation of learning methods

iRFFRR was tested comparatively against standard (linear) RR and KRR, both in batch and incremental modes, when possible—to this aim, notice that RR and iRFFRR do support incremental training, whereas KRR does not. For iRFFRR, we used 1000 RFFs (denoted as iRFFRR^1000^), which was found to give a reasonable tradeoff between accuracy and computational requirements. The motivation for including KRR was that it allows to determine the accuracy of the RFF approximation with respect to the full kernel approach. Standard (linear) RR, on the other hand, was included to investigate whether the non-linearity provided by the RFF mapping was indeed necessary, or whether a standard linear model would suffice. All methods were trained to jointly predict outputs for all five DOFs using the efficient method described in section 2.2.1.

The iRFFRR algorithm requires configuring the regularization parameter λ and the RFF parameter γ. The latter corresponds to the same parameter of the (approximated) RBF kernel that controls the bandwidth. In the following experiments, both hyper-parameters were optimized using *k*-fold cross-validation, where each of the folds corresponds to exactly one of the trials. As argued by Gijsberts et al. (2014), this particular assignment of the folds ensures that distributional differences among trials are taken into account when optimizing the hyper-parameters. The parameters were selected from a dense grid search with λ ∈ {2^−12^,2^−11^,…,2^5^} and γ ∈ {2^−8^,2^−7^,…,2^7^}. Note that the number of RFFs *D* does not require tuning; since the accuracy of the kernel approximation improves with increasing *D*, it suffices to choose *D* as large as computationally affordable.

Additionally, we also tested the performance of iRFFRR alone when trained in three different ways, namely on full sensor values, on full stimulus values and on binary stimulus values, that is, on resting ad maximal stimulus values only. All in all, three “settings” were chosen.

***2.3.3.1. Batch setting***. In the batch setting we considered a traditional batch learning approach, in which methods are trained on full sensor values at the beginning of the experiment (first three trials of the first session). We compensated for a constant bias in the output values (i.e., the intercept) by subtracting their mean value in the training data. Subsequently, the λ and γ hyper-parameters (when applicable) were optimized using threefold cross validation, where each fold corresponds to one of the training trials. To reduce computational requirements, the data used for hyper-parameter optimization were regularly sub-sampled by a factor of 4 (i.e., a window increment of four samples rather than one). The identified optimal parameter configuration was used to train a model on data from all training trials, while the remaining trials were used in their original order to test the model. Though not the primary scope of this paper, this batch setting gives insight on the amount of degradation that is present in the acquired data. To distinguish between inter- and intrasession degradation, we also considered an alternative batch setting in which the methods are trained from scratch on the first three trials *of each session*.

***2.3.3.2. Incremental setting***. In the incremental setting, iRFFRR^1000^ and linear RR were initially trained on the first three trials of the first session, that is, identically to the batch setting, but were, however, allowed to perform an incremental update on each subsequent first and tenth trial of all sessions, while the other trials were instead used for testing. Also in this setting the trials used for training and testing were processed in their original order, thus ensuring that the model used to test a sample at time *t*_test_ had not been trained on any future samples (i.e., *t*_train_ < *t*_test_). Violating this condition, for instance by shuffling the data, leads to a setting that does not correspond to reality and obfuscates the effect of distribution shifts over time.

***2.3.3.3. Realistic settings***. In the former two settings, models were both trained and tested on data recorded by the force sensor. From a practical point of view, these settings are of limited interest, since force measurements are not available from amputees; moreover, one cannot expect amputees to produce precisely graded forces, as they have no proprioception left (or in the extreme case, they have false proprioception induced by the phantom feeling). Following recent suggestions by Sierra González and Castellini (2013), we replaced the force measurements during training either with the stimulus shown to the subject on the screen (realistic setting 1), or with a binary version of the stimulus, obtained by thresholding at 50% MVC (realistic setting 2). This latter setting represents the most realistic possible setting, in which amputees are only requested to rest or flex one finger in turn. Note that testing was, however, still done on the measured forces, in order to compare the prediction with the ideal ground truth.

#### 2.3.4. Performance measures

Performance was evaluated using two measures, namely the normalized Mean Squared Error (nMSE) and the Pearson correlation coefficient (henceforth simply correlation). The nMSE is defined as the MSE normalized by the variance of the true output values and relates directly to the *R*^2^ measure as *R*^2^ = 1 − nMSE. To ensure an identical error measure in both batch and incremental settings, we used the variance calculated over all of a subject's trials as normalization factor rather than over only the test trials. The definition of the nMSE allows for an intuitive baseline result, since the trivial regressor that constantly predicts the mean output value (i.e., 0 after intercept compensation) obtains nMSE = 1. In other words, nMSE ≥ 1 implies that learning was ineffective, while the inverse means that the model performs at least better than this trivial baseline.

The motivation for considering correlation as additional performance measure was that nMSE (or related error measures) does not necessarily reflect true control performance. Consider for instance a regression model that predicts values that deviate by a constant factor from the true outputs. This model will suffer a high nMSE, even though it would probably perform rather well in practice (cf. the user could easily compensate for the deviation). Correlation, on the other hand, is insensitive to such constant deviations and measures more directly whether the force predictions correspond in some manner to the intended forces.

### 2.4. Experiment 2: demonstration

#### 2.4.1. Experimental protocol

One of the experimenters (CC) wore the sEMG setup around the forearm (similarly to what can be seen in Figure 1B) and the magnetic tracker sensor slightly down the forearm, attached on top of a velcro strap; a custom-made, light orthosis was worn around the wrist and fingers of the operator to provide resistance when producing the required forces while freely moving the hand and arm. No force sensor was used.

An initial training phase was then started. Three standard grasping configurations, namely pinch-gripping (full flexion of thumb and index, all other fingers at rest), power grasping (all fingers flexed) and pointing (all fingers flexed except the index), plus the resting state were used as training patterns. Notice that, even though the underlying method was predicting forces for all five fingers, it was tuned toward these multi-finger configurations simply by means of the targeted training procedure. Additionally, flat grasping (e.g., to grasp a credit card) was implemented by applying a power grasp with the prosthetic thumb fully opened. This motion was realized by manually moving the thumb of the prosthesis, as is required in real life with this particular prosthetic hand.

After the initial training phase, the operator was engaged in 20 repetitions of four tasks, each one subdivided into phases (please also refer to the movie in the Supplemental Material):

1. *Pick and place a ball* (3 min 25 s into the movie): the subject (1) reached for the yellow ball on the base; (2) picked it up with a pinch grip and put it on the wooden container; (3) returned to the start position and reached again for the ball; (4) picked it up again with a pinch grip and put it on the metal base; (5) returned to the start position.
2. *Pick and place a ball* (3 min 25 s into the movie): the subject (1) reached for the yellow ball on the base; (2) picked it up with a pinch grip and put it on the wooden container; (3) returned to the start position and reached again for the ball; (4) picked it up again with a pinch grip and put it on the metal base; (5) returned to the start position.
3. *Drink from bottle* (3 min 56 s into the movie): the subject (1) reached for a glass bottle on the base; (2) grabbed it with a power grasp, brought it close to the robot's head and mimicked a drinking movement by pronating and supinating the wrist, then put it back on the base; and (3) returned to the start position.
4. *Push button* (4 min 15 s into the movie): the subject (1) reached a yellow button on the base; (2) pressed it with a pointing index finger, reached and pressed another button on the vertical wall, and then pressed the first button again; (3) returned to the start position.
5. *Take credit card* (4 min 30 s into the movie): the subject (1) reached for a credit card inserted into a slit on the wall; (2) extracted it with a flat grasp and handed it over to a human operator; (3) returned to the start position.

The four tasks were designed to (a) require large reaching movements in the workspace, involving large translations and rotations of the end-effector (pronation/supination of the wrist) both while grasping and not grasping, and (b) to employ all possible grasp types in a meaningful way. Task 1 was meant as a test for the pinch-grip, requiring a stable grasp during phases 2 and 4. Task 2 tested the stability of a power grasp used to drink from a bottle while pronating and supinating the wrist. Task 3 tested the stability of a pointing action in two different arm and hand configurations (the two buttons were placed in two completely different positions and orientations); lastly, Task 4 tested the stability of the flat grasp (stable grasp required during phase 2). The experiment lasted in total slightly over 75 min.

#### 2.4.2. Feature extraction

In this experiment the data obtained from the surface electromyography electrodes were used directly, without applying any windowing, in order to reduce the delay in the teleoperation scenario. (The signal obtained from the electrodes used in this work has already been proved effective in literature Castellini and van der Smagt, 2009; Castellini et al., 2009).

#### 2.4.3. Learning method

The iRFFRR was the only method used in this experiment, since it is incremental and gave good results during the algorithm evaluation. After an initial round of experiments, 300 RFFs were used, which provided a reasonable balance between computational cost and prediction accuracy. Furthermore, we fixed λ = 1.0 and γ = 2.47; these values lied in the range of optimal values found during the Algorithm evaluation experiment. The training phase was performed according to the second realistic setting. The training data were collected with the operator performing only two trials of the grasp configurations in a relaxed position (right arm leaning down, shoulder relaxed), such that the training phase lasted a mere 54 s.

#### 2.4.4. Performance measures

The success of each task was assessed at the end of each repetition. A repetition was considered successful if the operator went through all phases without dropping the object; regrasping (i.e., losing the object but immediately grasping it again without letting it fall) was on the other hand considered acceptable. The data obtained during the experiment were manually segmented by detecting the onset and end of each successful repetition. An increase above 10 cm/s of the end-effector velocity would indicate the onset, whereas a drop below this value would denote the end. The task completion time was then determined based on these two segmentation points. Within each repetition, the duration of stable grasping was calculated using a threshold of 0.3 a.u. on the grasping force prediction. Based on the determination of onset and end of grasping, the phases within each task were identified. To quantify the stability of each of these phases, we evaluated the range of motion in the three dimensions (difference between minimum and maximum position reached by the end-effector during all tasks), the maximum speed along the trajectory, and (in particular for task 2) the angular range and the maximum angular velocity of the wrist rotation.

## 3. Results

### 3.1. Algorithm evaluation

#### 3.1.1. Batch setting

**Figure 3:**
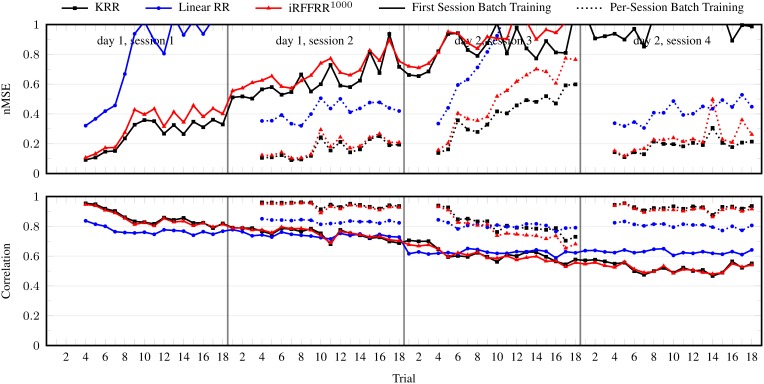
**nMSE and correlation coefficient per trial, in the batch setting: when training on the first three trials of the first session, and on the first three trials *per session***. Results for the linear RR and KRR methods are averaged over the ten subjects and the five DOFs. For the iRFFRR method, the results are in addition averaged over 25 runs with different random initializations.

The nMSE and correlation per trial averaged over subjects, DOFs, and 25 randomized runs (for iRFFRR) are shown in Figure 3. Although the performance was excellent on the first trials after training, it degraded almost instantaneously and monotonically. During the second session the performance was already close to nMSE = 1, meaning that the model had become highly ineffective. Retraining a new model from scratch on the first three trials of each session reset the performance to the initial level after training and was effective at counteracting degradation. Regardless, there still remained a gradual but steady performance decrease within a single session, which was most profound in the first session of each day.

**Figure 4:**
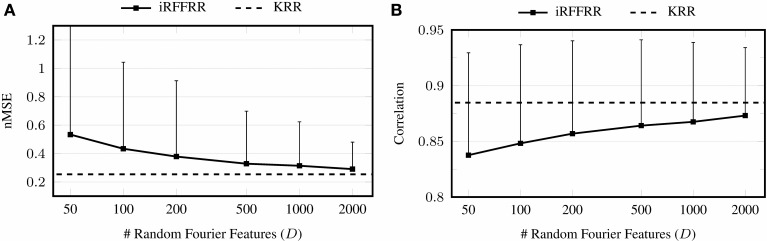
**Convergence of performance in terms of nMSE (A) and correlation (B) when increasing the number of RFFs *D***. The error bars indicate the intrasubject standard deviation over the 25 runs with different random initializations, averaged over the ten subjects and over the five DOFs.

Linear RR performed considerably worse than KRR and iRFFRR^1000^ in either batch setting. Noting specifically that its performance was also worse directly after training (e.g., trial 4 of each session); this confirmed the hypothesis that linear models do not have sufficient capacity to model the relationship between sEMG and multiple finger forces. The performance of iRFFRR^1000^, on the other hand, was very close to KRR, both in terms of nMSE as well as correlation. Further insight on the quality of the approximation was provided by Figure 4, which showed that iRFFRR converged monotonically to KRR with an increasing number of RFFs. This convergence related both to the average performance as well as to a reduction in the variance among different random initializations. This latter effect was less pronounced for the correlation, because iRFFRR by definition minimizes the MSE instead (see Equation 1). Improvements in terms of correlation were therefore a side-effect rather than a direct goal of the algorithm.

#### 3.1.2. Incremental setting

**Figure 5:**
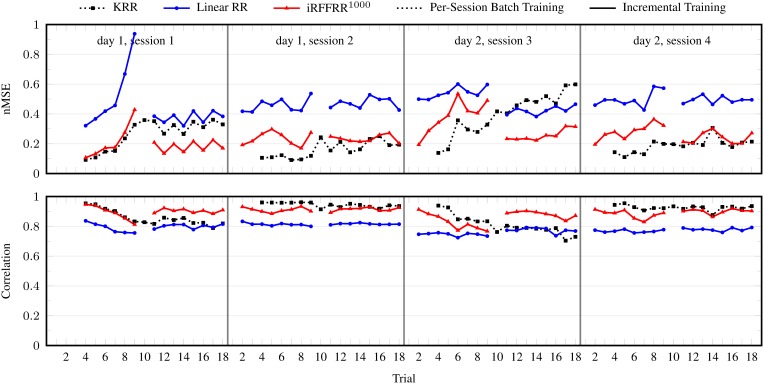
**nMSE and correlation coefficient per trial, in the incremental setting**. For comparison, the results for KRR trained per session are also included. The presented results for the linear method are averaged over the ten subjects and the five DOFs. For the iRFFRR method, the results are in addition averaged over 25 runs with different random initializations.

Figure 5 shows the results for the incremental setting. The figure also includes batch KRR retrained on the first three trials of each of the four sessions (see previous section), for comparison. It is clear from the figure that updating on a single trial was effective at correcting performance degradation. Overall, iRFFRR^1000^ performed roughly the same as the batch KRR method, even though the incremental method had fewer training data in total (i.e., 3 + 1 + 3 × 2 = 10 trials versus 3 × 4 = 12 trials). In particular during the first and third session, performance increased considerably after the incremental update on the tenth trial.

#### 3.1.3. Realistic settings

**Figure 6:**
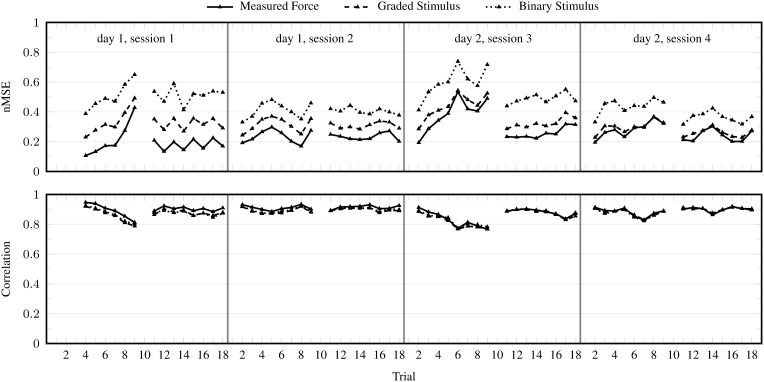
**nMSE and correlation coefficient per trial obtained by iRFFRR^**1000**^, in the incremental setting (training on measured forces) and in the realistic setting 1 (training on graded stimulus) and 2 (training on binary stimulus)**. The results are averaged over 25 runs with different random initializations.

Figure 6 shows the performance of iRFFRR in the realistic setting 1 and 2. Not surprisingly, the nMSE increased considerably when training on the stimulus instead of actual forces, particularly in case of the binary variant. Regardless, the overall performance over all test trials was nMSE ≈ 0.464, which was still considerably better than the baseline. This result is roughly similar to the performance reported by Sierra González and Castellini (2013) when predicting finger forces from ultrasound in a similar realistic setting. For comparison with their results, our nMSE ≈ 0.464 corresponds to a normalized Root Mean Square Error of approximately 0.167.

**Figure 7:**
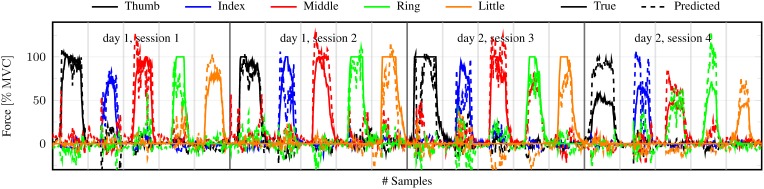
**Typical measured and predicted forces by iRFFRR^**1000**^ for the last trial of each session in the realistic setting 2 (training on the binary stimulus)**. Data taken from the first randomized run of the fifth subject.

The performance difference when using the stimulus for training rather than graded forces was much less pronounced in terms of correlation. The sample force measurements and predictions in Figure 7 are instrumental in understanding this observation: the figure shows inconsistencies in the force activations produced by the subject, ranging from approximately 40–100% MVC. These variations in force activations were not present in the stimulus, thus causing difficulties for the stimulus trained method to correctly estimate the magnitude of the activation. Regardless, these methods were generally able to reliably identify which finger was activated, as is evident from the correlation of almost 0.9 between predictions and actual measured forces.

**Figure 8:**
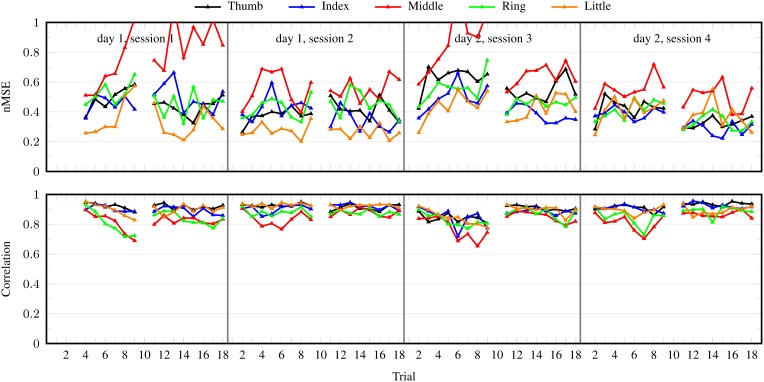
**nMSE and correlation coefficient per trial for individual DOFs obtained by iRFFRR^**1000**^ in the realistic setting 2 (training on the binary stimulus)**. Results averaged over the 10 subjects and over 25 runs with different random initializations.

Lastly, Figure 8 shows the performance per DOF of iRFFRR^1000^ when trained on the binary stimulus (realistic setting 2). The behavior was roughly similar for all DOFs, although the performance was worse for the middle finger.

### 3.2. Demonstration

**Figure 9:**
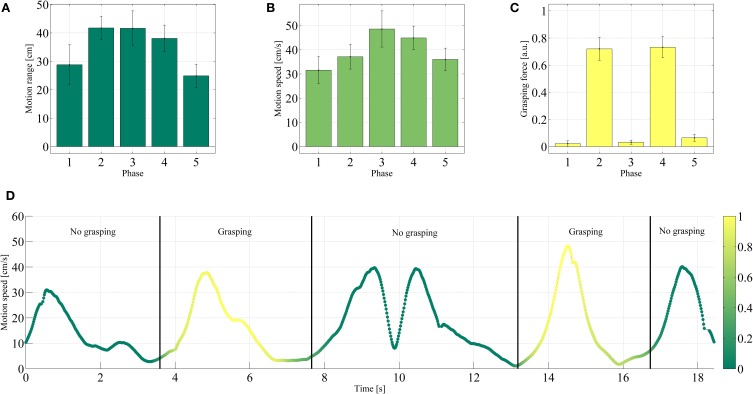
**Comparison of the end-effector motion range **(A)**, motion speed **(B)**, and grasping force **(C)** of the end effector during each phase of Task 1**. The mean values and one standard deviation are reported over the successful trials. **(D)** Motion speed during one typical trial. The color denotes the grasping force in arbitrary units, according to the color bar on the right-hand side.

**Figure 10:**
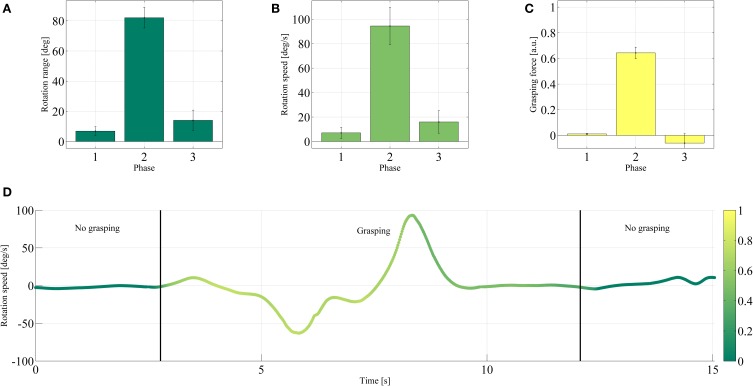
**Comparison of the wrist rotation range **(A)**, wrist rotation speed **(B)**, and grasping force **(C)** of the end effector during each phase of Task 2**. The mean values and one standard deviation are reported over the successful trials. **(D)** Rotation speed during one typical trial. The color denotes the grasping force in arbitrary units, according to the color bar on the right-hand side.

The results obtained during Tasks 1 and 2 of the demonstration are shown in Figures 9, 10, whereas Tables 1, 2 give an overview of the whole procedure. Tasks 1 and 2 have been selected for the figures since these are the most complex tasks and therefore the most interesting. Particularly Task 1 required two distinct stable grasps separated by a non-grasping phase. In contrast, Task 2 stressed the resilience of the power grasp with respect to wrist rotation.

**Table 1:** **The success rate and duration for all four tasks and the duration, motion range and speed, and grasping force (in arbitrary units) for each phase of each task**.

**Task**	**Phase**	**Duration [s]**	**Range [cm]**	**Speed [cm/s]**	**Force [a.u.]**
*Task 1*	1		28.82±7.05	31.56±5.50	0.02±0.02
Success: 16/20 (80%)	2	5.07±0.89	41.76±4.12	37.12±5.12	**0.72 ± 0.08**
Duration: 22.32 ± 319 s	3		41.64±6.12	48.57±7.47	0.03±0.02
	4	4.53±0.94	38.09±4.66	44.88±4.88	**0.73 ± 0.08**
	5		24.91±4.10	36.01±4.65	0.06±0.03
*Task 2*	1		29.99±6.00	26.97±5.46	0.01±0.00
Success: 15/20 (75%)	2	9.09±1.04	21.39±8.66	57.91±6.37	**0.64 ± 0.04**
Duration: 15.44 ± 354 s	3		29.73±4.70	33.20±5.21	−0.06±0.07
*Task 3*	1		22.73±17.63	27.52±14.69	0.04±0.04
Success: 18/20 (90%)	2	13.96±3.94	51.46±7.92	37.77±6.60	**0.71 ± 0.07**
Duration: 16.82 ± 501 s	3		19.30±11.05	29.01±9.55	0.08±0.04
*Task 4*	1		45.45±4.15	28.57±5.77	0.01±0.02
Success: 19/20 (95%)	2	5.05±1.27	30.38±5.51	27.13±4.34	**0.64 ± 0.08**
Duration: 11.70 ± 175 s	3		42.75±11.57	41.12±8.19	0.02±0.02

*The mean value and standard deviation refers to the successful repetitions. Furthermore, boldface denotes the force values in phases that involved grasping*.

**Table 2:** **Angular range [◦] and speed [◦/s] for each phase of Task 2**.

**Phase**	**Range [◦]**	**Speed [◦/s]**	**Force [a.u.]**
1	6.88 ± 2.99	7.07 ± 4.49	0.01 ± 0.00
2	82.08 ± 6.82	94.47 ±15.21	**0.64 ± 0.04**
3	14.05 ± 6.75	15.97 ±9.16	−0.06 ± 0.07

*The mean value and standard deviation refers to the successful repetitions. Furthermore, boldface denotes the force values in phases that involved grasping. For convenience, the force values from Table 1 are repeated*.

Consider Figures 9A–C, showing the end-effector average motion range, motion speed and grasping force for each of the five phases. Both the range of motion (24.91–41.76 cm) and speed (31.56–48.57 cm/s) were relatively large in all phases and compared to the size of the reaching/grasping setup, accounting for reaching phases in the order of magnitude of 1 s. As opposed to that, the average grasping force was lower in the phases that did not involve grasping (phases 1, 3, and 5, 0.02–0.06 a.u.) and larger in the ones that did (phases 2 and 4, 0.72 and 0.73 a.u.). This behavior can also clearly be observed in Figure 9D, which shows how the motion speed and grasping force progressed over time for a typical successful repetition of Task 1.

Figures 10A–C depicts similar results for the angular range and speed during Task 2, relative to wrist pronation and supination. The angular range and speed were larger during the grasping phase when the wrist rotation was required (phase 2, 82.08° and 94.47°/s) than when it was not (phase 1, 6.88°, 7.07°/s and phase 3, 14.05°, 15.97°/s—see again the movie in the Supplemental Material, from 3 min 56 s on). Similar to what happened during the previous task, the average grasping force during phase 2 (0.64 a.u., when the drinking action took place) was found to be higher than during the other phases (phase 1, 0.01 a.u. and phase 3, −0.06 a.u.—this small negative force is due to sEMG artifacts). Figure 10D shows the wrist rotation speed during a typical successful repetition of Task 2.

The results shown in Tables 1, 2 confirm the stability of grasping in all tasks and phases: for each task, the average grasping force was much higher during phases that actually involve grasp actions. This indicated that the control system was correctly recognizing the high force commands typical during grasps, and that this capability remained stable throughout the entire experiment. The tables also show that the success rate of these common activities was overall high, ranging between 75% for Task 2 and 95% (Task 4).

## 4. Discussion

The results presented in the previous section show the effectiveness of iRFFRR in predicting finger forces and force grasping patterns from surface electromyography: the prediction is *accurate* and *stable*. Moreover, the system can be implemented to work at a rate suitable for practical use. Taking into account that the electrodes and the mechanical hand we have used are off-the-shelf commercial products, both routinely used in the clinical practice, it is safe to claim that this system could be used in practice to naturally and reliably control a dexterous hand prosthesis.

### 4.1. Algorithm evaluation

Consider Figure 6, where the performance of iRFFRR is shown in the incremental setting as well as in the two realistic settings. Although—as expected—the performance got worse and worse from the incremental setting to the two realistic settings, even in the second realistic setting it stayed almost always within 0.3–0.6 nMSE, an error comparable to that found, e.g., in Sierra González and Castellini (2013). Incremental training was effective in keeping the performance high; for instance in session 3, retraining on the tenth trial improved the nMSE from 0.7 (ninth trial) to 0.45 (eleventh trial); on the other hand, the necessity of a non-linear method is clearly shown by the comparison between iRFFRR and both RR and KRR reported in Figures 3, 5: RR obtained a consistently worse performance than iRFFRR and KRR, mostly unacceptable (close to 1). Since KRR is not incremental, iRFFRR represents an accurate *and* practically usable solution to this problem.

In the incremental and realistic scenarios, the first and tenth trial of each session were treated as training data, meaning that (excluding the initial batch training) one out of nine trials was used for training. An important practical advantage of this incremental procedure was that it allowed to distribute the training effort more uniformly in time, as opposed to the concentrated effort required for batch (re)training. Each re-training phase required the user to flex the fingers, for instance by pressing the finger on a table—recall that the realistic scenarios are sensorless, since the ground truth is obtained from the visual stimulus. This procedure, which takes approximately 12 s per finger, works independently finger by finger and could be activated at any time, meaning that as soon as the subject deems the precision for a certain finger insufficient, (s)he can simply ask for re-training.

Moreover, the second realistic setting represented a realistic scenario for amputees, who obviously cannot operate any force sensor and are generally unable to perform graded tasks due to the lack of visual and proprioceptive feedback; the latter is, in many cases, even inconsistent with the intended action (there is ample literature on the subject of phantom feelings; see, e.g., Diers et al., 2010). In our system on the other hand, the subject could be instructed to just rest and maximally flex the selected finger when required, a much simpler operation.

Notice that in the two realistic settings, i.e., when the visual stimulus values rather than the sensor values were used as ground truth, the performance was always evaluated *by testing on the fully graded range of the force sensor values*, that is, on the “ideal” ground truth. Since the performance was comparable in this setting too, we claim that the method was correctly interpolating the intermediate values, enabling accurate results with minimal and easy training. This result is similar to that obtained using ultrasound images in Sierra González and Castellini (2013), in which RR could be proficiently used since the relationship between features of the ultrasound images and finger forces is linear (Castellini and Passig, 2011).

One essential question is: how fast can the system operate in practice? In the configuration used in the experiments with 1000 RFFs, iRFFRR required approximately 30 ms per update utilizing only a single core of a modern Intel i7 CPU, while predictions took around 100 μs. Memory consumption instead is dominated by the *D* × *D* covariance matrix, which requires 8 MB assuming *D* = 1000 and double precision floating point representation. Moreover, updates and predictions of iRFFRR are characterized by a constant time and space complexity; this means that the computation time and memory consumption remain constant regardless of the number of previous training samples. Boundedness and predictability of the computational requirements are important features that allow open-ended use in a (hard) real-time setting, for instance on embedded hardware. A further practical advantage is that the computational requirements can be lowered arbitrarily by utilizing fewer RFFs, at the cost of decreased performance (cf. Figure 4). All in all, the practical viability of this system, at least in this experiment, is assured.

### 4.2. Demonstration

During the teleoperation experiment, an expert intact subject could reliably perform a repetitive teleoperated picking and placing experiment using the same method described in section 2.2. The training phase lasted less than 1 min and enabled reliable grasping over 1 h and a quarter; precise Cartesian positioning of the robotic end-effector was enforced using impedance control with high stiffness. The grasping remained stable, both when required and when *not* required, notwithstanding wide changes in the operator's arm kinematic configuration (large motion range) and wrist pronation/supination (large rotation range).

During the demonstration no re-training was necessary; we deem that this was due to the relatively long time allowed for the electrodes to warm up with respect to the subject's skin. In fact, inspecting more closely the long-term results obtained during the algorithm evaluation (consider Figure 3 again), one sees that the degradation in performance is worse along sessions 1 and 3; since these sessions are the two in which the subjects started afresh, it seems reasonable to claim that the degradation was mainly due to a factor affecting the very early trials, the best candidate being the adaptation of the electrodes to the skin temperature. Temperature differential between electrodes and skin can actually cause relevant changes in the signal itself (Winkel and Jørgensen, 1991).

Notice, furthermore, that for the demonstration the system was trained with force grasping patterns rather than with single-finger patterns as it happened in the algorithm evaluation. This was necessary since grasping consists of simultaneous multi-finger force patterns, and it is as yet unknown how to predict them by training on single-finger force patterns only. Notice, however, that the only difference between the system used in the two experiments lies in the way it was trained: even during the demonstration, the predicted output always consisted of *five graded values representing the forces at the fingertips*. This goes in the direction of practical enforcement of natural control, avoiding the restriction, typical of classification, of predicting one among a finite, usually small set of possible kinematic configurations.

Lastly, notice that we intentionally used two different performance measures for the algorithm evaluation and for the demonstration: nMSE and correlation in the first case, task completion success ratio and stability of the grasp in the second. The motivation for this lies in the necessity of providing a practical measure of performance, e.g., “how many times would the prosthesis let a mug full of coffee fall to the ground?,” as opposed to abstract measures of performance, which risk to be meaningless in real life (for a discussion on this point see, e.g., Wagstaff, 2012).

## 5. Conclusion

Prosthetic myocontrol is known to be unreliable due to changes in time in the myoelectric signal, caused by perspiration, electrode displacement, fatigue, kinematic configuration of the arm, etc. In this paper we proposed to enforce stable, dexterous force myoelectric control by means of a non-linear incremental method: non-linearity enforces accurate prediction and incrementality guarantees adaptation to changing conditions: the system works even in a realistic scenario where no sensors need be used to gather the ground truth. A detailed evaluation was presented to substantiate these claims, as well as a practical demonstration, in which a robotic arm equipped with a prosthetic hand is teleoperated to grasp objects in a controlled setting. Incremental retraining was not tested during the demonstration, as it turned out not to be required.

### Conflict of interest statement

The authors declare that the research was conducted in the absence of any commercial or financial relationships that could be construed as a potential conflict of interest.
